# Extensive guttate morphea in an adolescent

**DOI:** 10.1016/j.jdcr.2026.09.029

**Published:** 2026-10-17

**Authors:** Vera Wang, Kevin Mai, Catherine Tawfik, Aurel Apple, Paul Chu, Cindy Hoffman, Charles Gropper

**Affiliations:** a College of Osteopathic Medicine of the Pacific, Western University of Health Sciences, Pomona, California; b Department of Dermatology, St Barnabas Hospital, Bronx, New York; c Bridge Dermpath-Tarrytown, Tarrytown, New York

**Keywords:** Guttate morphea, morphea variants, pediatric, scleroderma

## Introduction

Morphea, also known as localized scleroderma, is an autoimmune inflammatory connective tissue disorder characterized by excess collagen leading to fibrosis of the skin and underlying soft tissue. It presents as patches of hardened, thickened skin mainly in the dermis and subcutaneous fat and may involve underlying tissue in severe cases.^1^

There are various classification schemes for morphea, all determined by clinical presentation, pattern, and depth of fibrosis. Guttate morphea is a rare variant of morphea characterized by small, scattered, well-demarcated patches of hardened, sclerotic skin with a whitish or violaceous hue, typically seen on the trunk and limbs. This subtype is limited to superficial dermal involvement, distinguishing it from deeper subtypes.^2^ Because of the rarity of guttate morphea, the prevalence and incidence are unknown.

The condition is diagnosed clinically and is often mistaken for other dermatologic conditions, leading to delayed diagnosis. Morphea shares histopathologic and clinical similarities with lichen sclerosus, and there are several reports of both coexisting, with a controversial relationship between the 2. Histopathology is often needed to distinguish them, as lichen sclerosus is hyperkeratotic with hydropic degeneration of the basal layer (vacuolar interface dermatitis) and demonstrates decreased elastic fibers, while morphea does not have elastic fiber involvement. Additionally, morphea does not involve the anogenital region, while lichen sclerosus usually does.^3^ Here, we present a rare case of guttate morphea in a 14-year-old male.

## Case presentation

A 14-year-old male presented to our clinic with a rash that started around a year ago. The patient notes that the rash started around his trunk before spreading across his trunk and bilateral extremities, sparing the hands and feet. The lesions were originally pruritic but became asymptomatic, appearing as skin-tinged white sclerotic papules with hyperpigmented brown borders (Figs 1 and 2). The patient denied any previous episodes of similar occurrences. He also denied any travel, sick contacts, or illnesses around the time of onset of his rash. No notable triggering events were noted, and the patient denied use of any new medications or recreational drugs around the onset of the rash. No Raynaud's phenomenon was appreciated on physical examination, and the patient denied any episodes of it in the past. An initial punch biopsy of the trunk noted a concern for morphea/scleroderma, with a second punch biopsy of the arm confirming the diagnosis. An elastic van Gieson stain was performed, which demonstrated unremarkable changes to elastic fibers, ruling out papular elastorrhexis (Fig 4). Serology workup was ordered, including ANA, SSB ab, Scl70 ab, anti-ssDNA, anti-smith ab, anti-Ro52, anti-Ro60, and anti-RNP ab, which were all unremarkable. The patient was treated with a trial of topical triamcinolone 0.1% ointment, with continued initial spread of the lesions before stabilizing over the next few months.

**Fig. 1:**
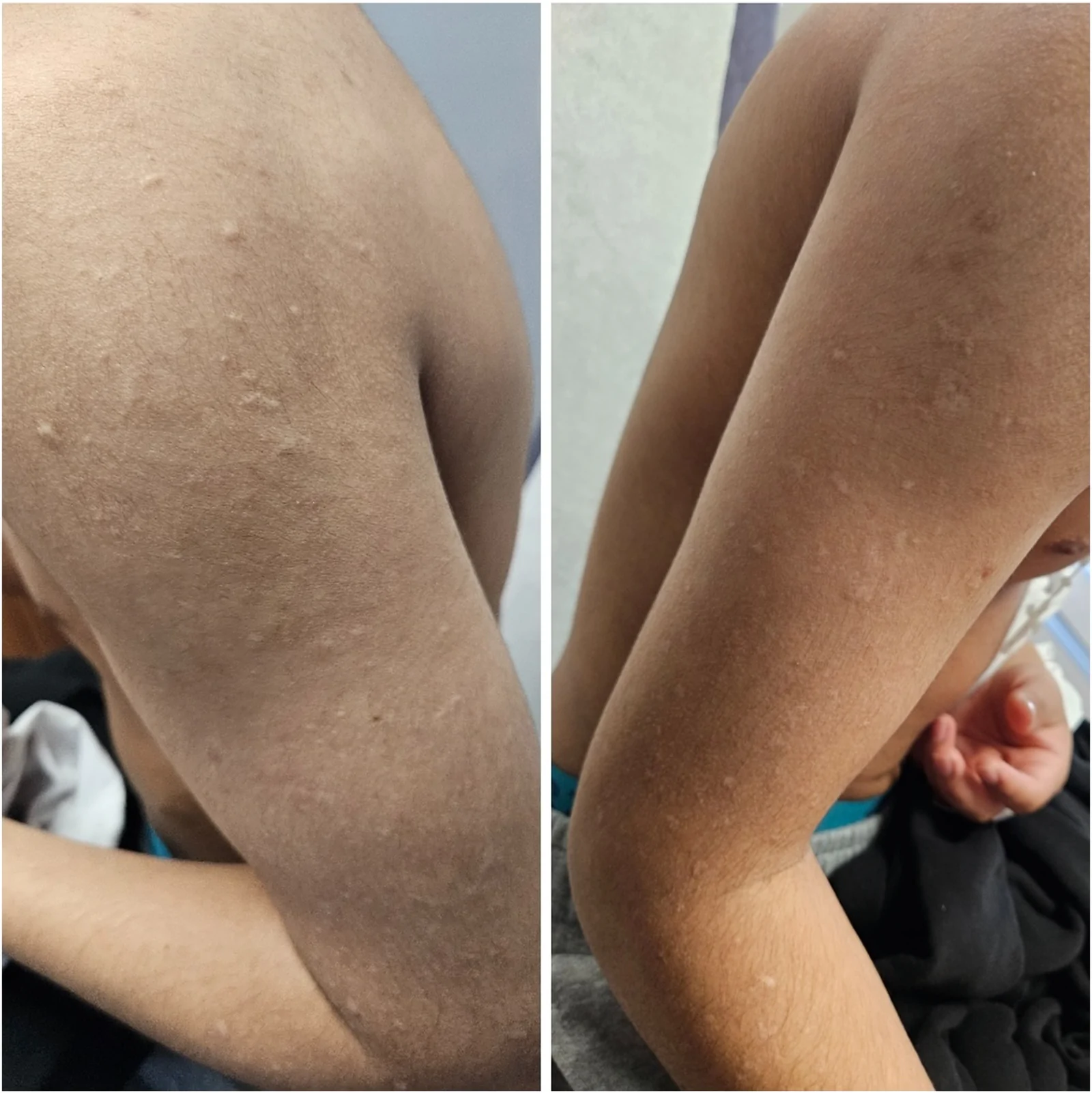
Skin-tinged white sclerotic papules with hyperpigmented brown borders on the bilateral arms.

**Fig. 2:**
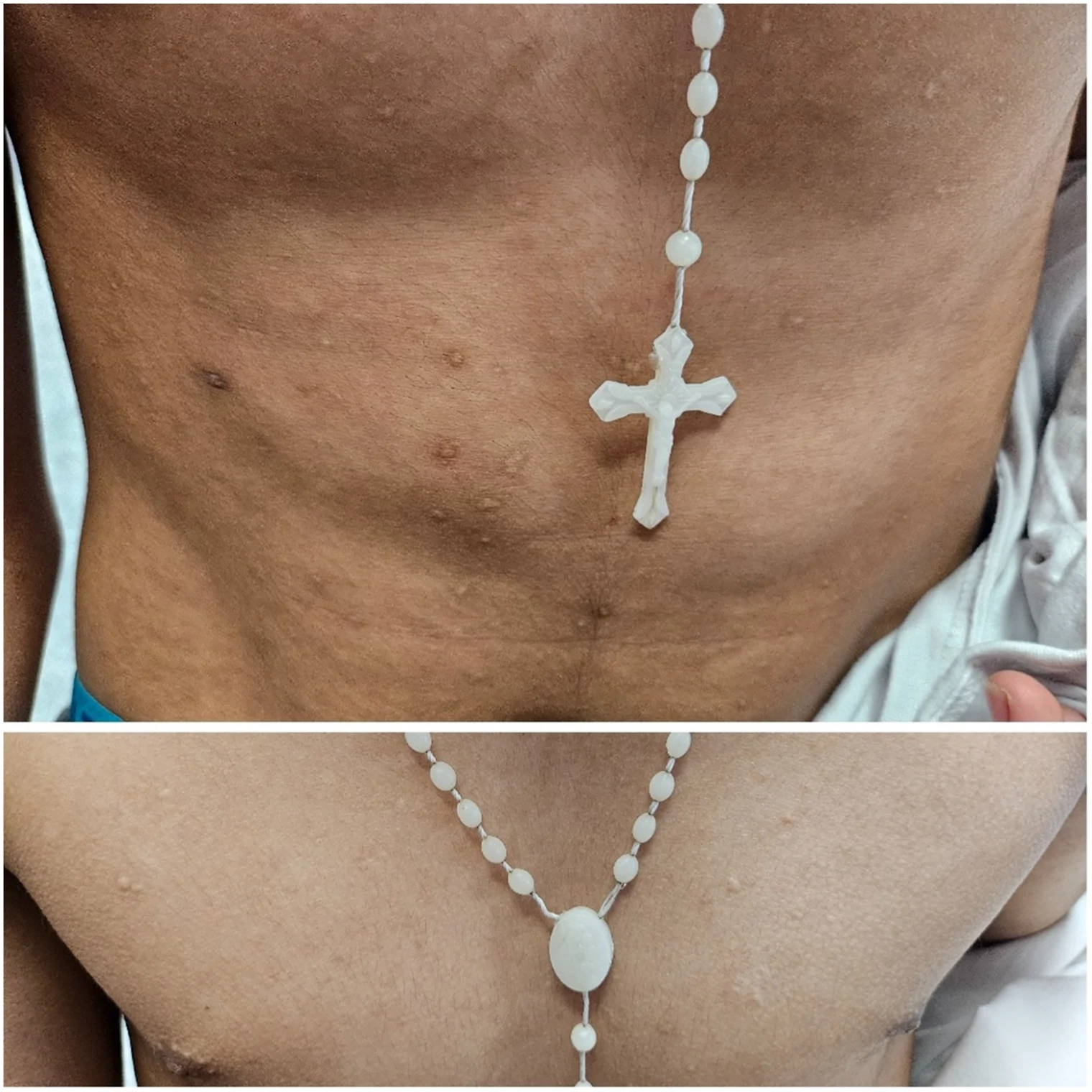
Skin-tinged white sclerotic papules with hyperpigmented brown borders on the anterior trunk.

**Fig. 4:**
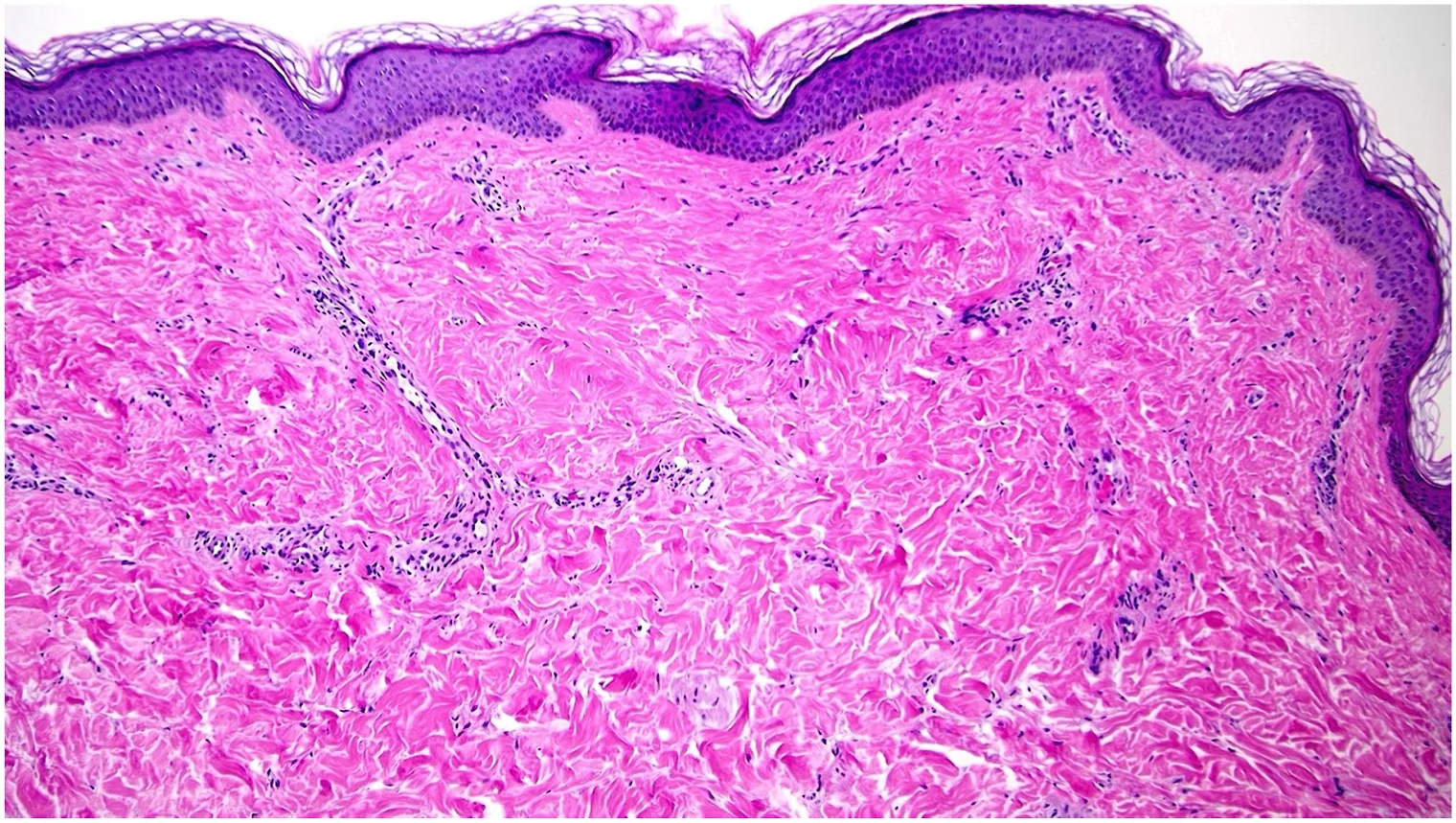
Higher power of the aforementioned punch biopsy on the arm highlighting the thickened sclerotic hyalinized collagen in the superficial dermis.

## Discussion

The pathogenesis of morphea is thought to involve genetic predisposition, immune dysregulation, and environmental triggers, but the exact mechanism is unclear. Hypoxia from microvascular injury is believed to be an early primary event, as it leads to altered cutaneous microvasculature, including a reduced number of capillaries. The resulting hypoxic environment triggers the activation of lymphocytes and disrupts normal connective tissue production, contributing to the development of fibrosis seen in morphea.^4^ Certain HLA class I and class II alleles have been associated with morphea, although these are only applicable to generalized and linear subtypes. Immune dysregulation plays a role throughout, with early stages likely driven by T-helper-1/T-helper-17 pathways, and later fibrotic stages driven by Th2 pathways.^5^ Also associated is an increased prevalence of anti-ssDNA, antitopoisomerase IIα, antiphospholipid, anti-fibrillin-1, and antihistone antibodies. Presence of ssDNA or antihistone antibodies in linear morphea is associated with increased risk of functional impairment. Current reviews recommend reserving autoantibody testing for patients with atypical features, extensive or rapidly progressive disease.

There are currently only 8 published cases of guttate morphea, mostly in females, and with only 1 pediatric case at age 7. Presentations included 2 cases with lesions on the flank area, 1 case with lesions on the buttock and thigh, and 1 more case on the upper extremities, hands, feet, and perioral region.^6^, ^7^, ^8^, ^9^, ^10^ Another case reported lesions on the chest and abdomen in a 44-year-old man, similarly to our patient; however, there was no further description of presentation.^10^ This entity holds similar overlapping clinical features with other cutaneous pathologies. When first encountering this presentation, a range of differentials were considered, including extragenital lichensclerosis et atrophicus, eruptive dermatofibromas, and eruptive anetoderma. During physical examination, no Raynaud’s phenomenon was appreciated, and the patient denied any episodes of such. The lack of finger involvement, such as sclerodactyly and nailfold capillary changes, also helped reduce the clinical suspicion of systemic sclerosis. The patient stated that many of these lesions first appeared on the abdomen. The possibility of a secondary scarring process to other primary lesions, such as acne, was raised. However, the patient and parent denied any history of acne or other morphologic variants of the lesion. The patient was treated with a course of topical corticosteroid after the initial biopsy, with the parents informed to take photographs of any new lesions. At a follow-up visit, it was noted that new lesions initially continued to erupt, which had similar clinical features to Figures 1 and 2, before the rash stabilized with treatment, which shifted the focus away from secondary postinflammatory changes. The histopathology further supports this, as a scarring process, such as that from acne scarring, would show organized collagen bundles with more fibroblastic presence as well as perpendicular vessels (Fig 3). Furthermore, the lack of elastic fiber changes in the histopathology allowed us to rule out papular elastorrhexis.

**Fig. 3:**
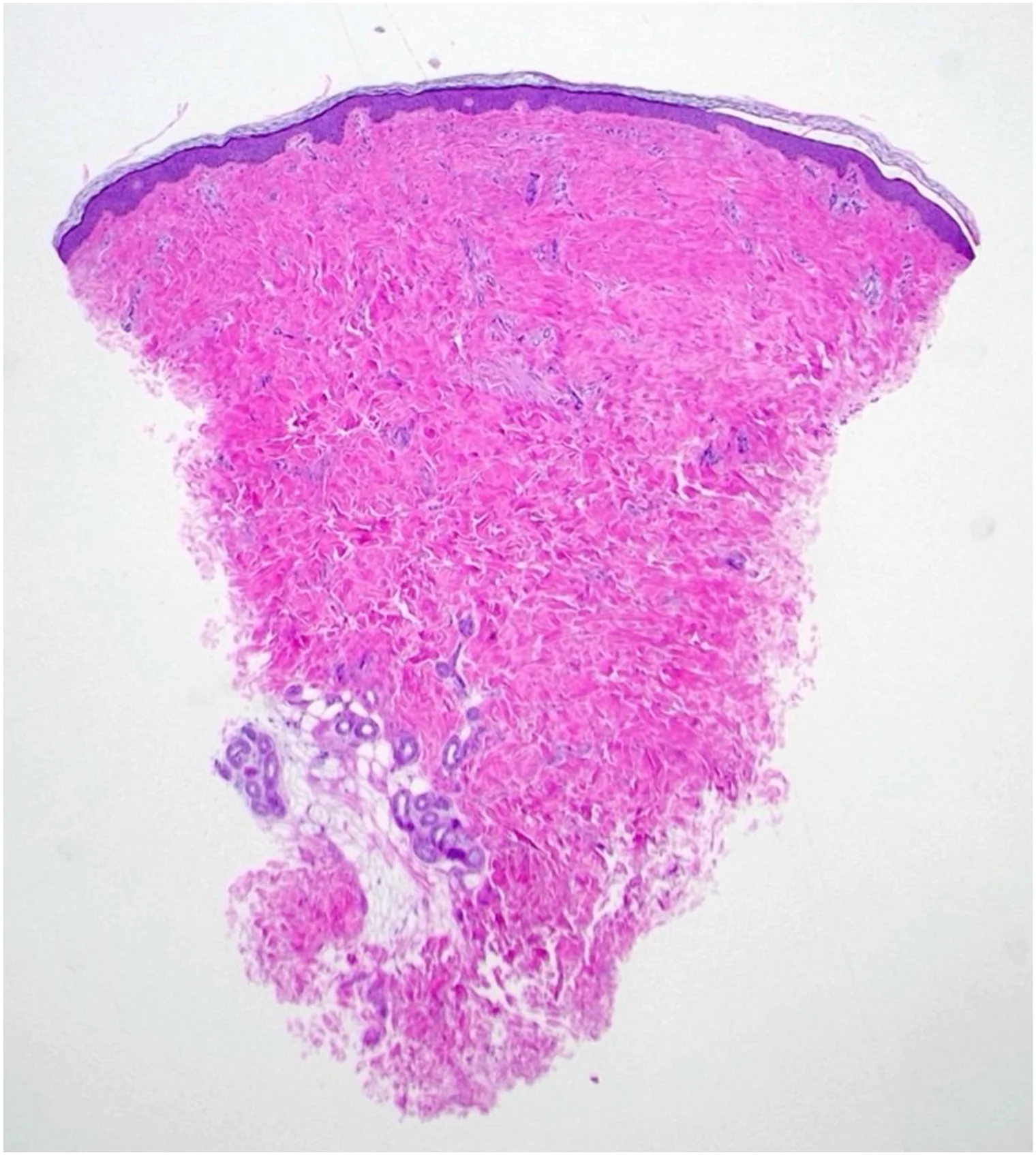
Low-power histopathology of a punch biopsy of the lesion on the arm demonstrating thickened sclerotic hyalinized collagen and loss of periadnexal fat extending from the superficial to mid dermis.

Behera et al trialed clobetasol ointment for the extremities and mometasone ointment for the face with resolution of itching but only minimal improvement of depigmentation.^7^ Other articles did not discuss treatment options.

Oiso et al report a patient with guttate morphea with positive human T cell lymphoma/lymphotropic virus type-1 (HTLV-1) and propose a possible association between guttate morphea and HTLV-1. HTLV-1 stimulates transcription of transforming growth factor beta 1 protein, which stimulates type 1 collagen gene promoters. Additionally, HTLV-1 upregulates interleukin-2, interleukin-2 receptor, interleukin-4, interleukin-6, and vascular cell adhesion molecule-1, all of which are elevated in patients with scleroderma.^9^

Overall, the location of guttate morphea in our patient has only been reported once, and there have been no reports of a patient within 6 years of our patient’s age with this condition. Additionally, the presentation of the lesions differs from that of all other cases, as our patient shows a greater percentage of body surface area affected and raised lesions that have not previously been described.

## Conflicts of interest

None disclosed.
